# Actinomycetoma Caused by *Gordonia rubripertincta*: Case Report

**DOI:** 10.3390/diagnostics16040509

**Published:** 2026-02-08

**Authors:** Rita Siksniute, Vilune Martinkiene, Rokas Jurkonis, Justinas Stucinskas, Kristina Valatkeviciene, Silvija Kiveryte, Kristina Marcinkeviciene, Danguole Vaznaisiene

**Affiliations:** 1 Department of Infectious Diseases, Lithuanian University of Health Sciences, LT-47116 Kaunas, Lithuania; 2 Department of Orthopaedics and Traumatology, Lithuanian University of Health Sciences, LT-50161 Kaunas, Lithuania; 3 Department of Radiology, Lithuanian University of Health Sciences, LT-50161 Kaunas, Lithuania; 4 Department of Physiology, Biochemistry, Microbiology and Laboratory Medicine, Institute of Biomedical Sciences, Faculty of Medicine, Vilnius University, LT-03101 Vilnius, Lithuania; silvija.kiveryte@santa.lt; 5 Laboratory of Microbiology, Centre of Laboratory Medicine, Vilnius University Hospital Santaros Klinikos, LT-08661 Vilnius, Lithuania; kristina.marcinkeviciene@santa.lt

**Keywords:** mycetoma, actinomycetoma, *Gordonia rubripertincta*, case report

## Abstract

**Background:** Mycetoma is a chronic infectious disease caused by bacteria or fungi which typically affects the skin, deep tissues, and bones. This case involves bone mycetoma in an immunocompetent patient, marking the first known instance of actinomycetoma caused by *Gordonia rubripertincta*. **Case Report:** A 25-year-old male presented with severe pain and deformity in his left foot, symptoms that began five years prior after stepping on a wire. Initial surgery provided temporary relief, but symptoms worsened over time. Doxycycline treatment was ineffective. Skin biopsies were performed. The patient was diagnosed with actinomycetoma, with *Gordonia rubripertincta* identified in culture. Although initial improvement was observed with amoxicillin–clavulanate treatment, the condition later worsened, requiring long-term penicillin therapy and eventual surgical excision. Despite treatment, symptoms persisted, leading to a bone biopsy that showed no microorganism growth. A six-week course of ampicillin–sulbactam and ciprofloxacin, along with offloading, decreased pain and stabilized radiological findings. **Conclusion:** *Gordonia* infections mean there is no universally established treatment protocol. This case underscores the diagnostic and therapeutic challenges associated with mycetoma, particularly in non-tropical regions.

## 1. Introduction

Mycetoma, also known as madura foot, is a chronic, granulomatous infection that affects the soft tissues and bones, typically in the feet. The condition is typically triggered by a minor injury that inoculates the causative microorganism into the subcutaneous tissue [1]. Mycetomas result from diverse species of fungi and bacteria, which typically thrive as saprophytes in soil or on plants. Actinomycetoma is caused by aerobic *Actinomycetes* from the *Nocardia*, *Streptomyces*, and *Actinomadura* genera, with *Nocardia brasiliensis*, *Actinomadura madurae*, *Actinomadura pelletieri*, and *Streptomyces somaliensis* being the most prevalent. Eumycetoma is linked to various fungi, with *Madurella mycetomatis* being the most common species [2]. Here we report a case of actinomycetoma caused by the bacterium *Gordonia rubripertincta*, an uncommon etiological agent of actinomycetoma. The information summarized in the Abstract is presented here in expanded detail [3].

## 2. Case Report

A 25-year-old male patient presented with deformation, pain, and severe swelling over the medial, dorsal, and plantar aspects of his left foot with a 5-year duration.

The symptoms began when the patient stepped on a wire. The patient underwent surgery, including incision and debridement, which initially improved his condition. However, the pain recurred after some time, and the affected area expanded, leading to progression of pain and deformation. The differential diagnosis for the condition included mycetoma, bacillary angiomatosis, Charcot foot, tuberculosis, atypical mycobacteriosis, deep mycosis, and abscess. Skin biopsies were performed several times, and the histological examination showed nonspecific changes, i.e., signs of chronic inflammation. Laboratory findings remained slightly altered: CRP (mg/L)—16.6 (reference range: 0–5 mg/L); WBC (×10^9^/L)—9.72 (reference range: 4.0–11.0 × 10^9^/L); ESR (using the Westergren method (mm/h))—34 (reference range: 1–20 mm/h). Treatment with doxycycline proved ineffective. The patient was suspected to have bacillary angiomatosis and erythromycin was prescribed. Itraconazole was also prescribed as eumycetoma could not be ruled out. Microbiological culture from skin lesions was performed. Small, slow-growing, orange-pigmented, non-haemolytic colonies were observed on 5% sheep blood agar, raising suspicion of *Actinomyces*. The colonies were catalase-positive and oxidase-negative. The causative organism was identified by matrix-assisted laser desorption ionization–time of flight mass spectrometry (MALDI BioTyper, Bruker, Bremen, Germany) as *Gordonia rubripertincta*. Anaerobic and fungal cultures were negative. Antimicrobial susceptibility testing was performed by the MIC test strip method (Liofilchem, Roseto degli Abruzzi, Italy). The antimicrobial agents tested included penicillin, amoxicillin–clavulanate and ciprofloxacin. EUCAST clinical breakpoints for non-species-related bacteria were used. The isolate was susceptible to penicillin, amoxicillin–clavulanate, and ciprofloxacin (see Table 1 for MIC values).

The treatment was changed to amoxicillin–clavulanate; initially there was improvement, but the condition worsened over time. Long-term penicillin treatment was prescribed, and surgical excision was performed due to negative dynamics on radiological tests (findings suggestive of progressive osteomyelitis and septic arthritis). Despite ongoing treatment, the symptoms persisted, with worsening swelling and pain, leaving the patient unable to fit their foot into a shoe.

The patient had not travelled to a tropical country. The patient had no comorbidities, and tests for diabetes, immunodeficiency (HIV test) and tuberculosis were negative.

**Figure 1:**
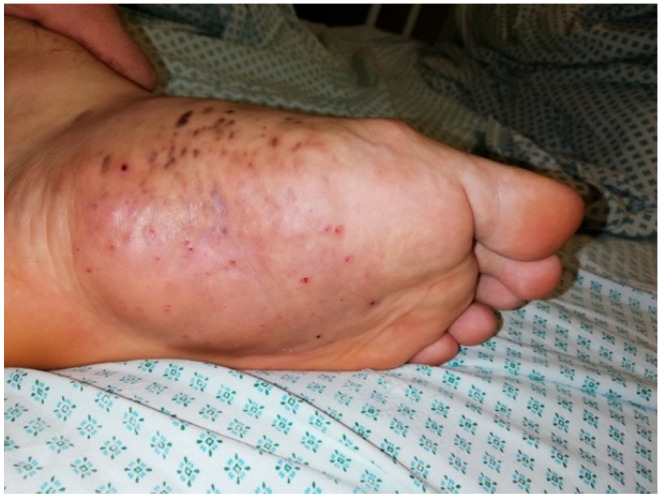
Foot photo before treatment.

Physical examination revealed left foot deformation, restricted range of motion, preserved sensations, tenderness on palpation, and a palpable hard mass on the plantar surface of the foot (Figure 1).

**Figure 2:**
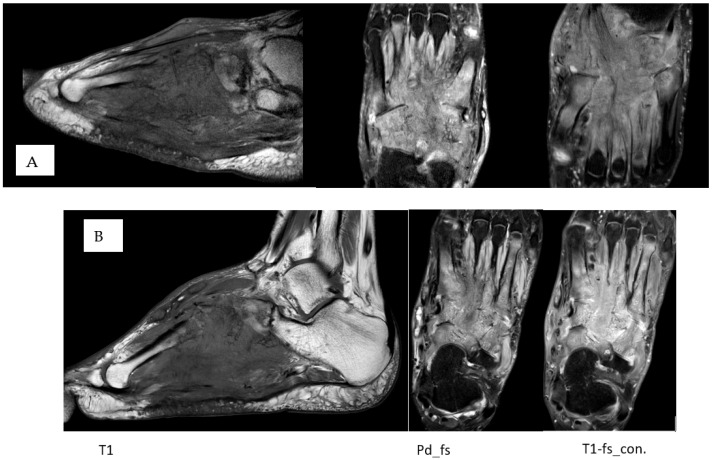
MRI before the bone biopsy (**A**) and repeated after treatment (**B**) showed that the findings remained stable.

The patient underwent a repeated MRI, which showed clear progression over an approximately 1.5-year period. Extensive, multiple coalescing small foci of osteomyelitis were present in the cuboid, navicular, all cuneiforms, the base of the V metatarsal, the larger part of the IV metatarsal, and the proximal side of the I-III metatarsals. These findings are characteristic of granulocytic damage. Additionally, there was associated septic arthritis in the naviculocuneiform, calcaneocuboid, and tarsometatarsal joints. A broad, diffuse, analogous small-grain inflammatory infiltration was observed in the surrounding soft tissues (Figure 2A).

A multidisciplinary decision prompted a bone biopsy, which was performed after at least a two-week antibiotic-free interval, which yielded no microbial growth. Treatment with 750 mg ampicillin–sulbactam twice daily and 750 mg ciprofloxacin twice daily was initiated for six weeks, along with offloading.

During the next visit, a repeated MRI was conducted, which showed no significant changes, indicating stable disease (Figure 2B). Over 2 years of monitoring, the patient’s foot pain and stiffness gradually decreased.

Following treatment, the patient reported a decrease in foot pain, with only minor discomfort occurring after prolonged walking. Clinical examination revealed no swelling, no progression of deformities, and the patient no longer experienced difficulties fitting the foot into shoes. The main remaining issue was a cosmetic deformity; however, the treatment successfully preserved the foot. Overall, the outcome was considered satisfactory, as the progression of destruction was halted and amputation was not required.

**Figure 3:**
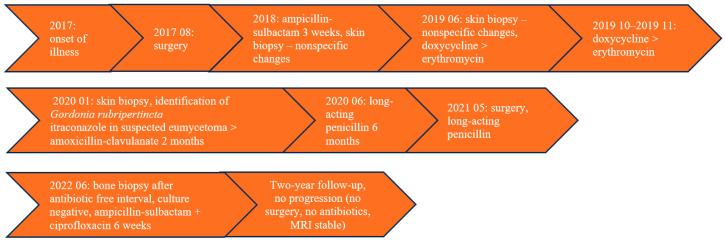
The timeline table summarizing the patient’s diagnostic and therapeutic course.

The timeline table summarizing the patient’s diagnostic and therapeutic course is presented in Figure 3.

## 3. Discussion

Mycetoma is a chronic, progressive inflammatory condition affecting the skin, the subcutaneous tissue, and the underlying bones. It is classified by the World Health Organization as one of the neglected diseases of tropical regions [1].

Mycetoma is most commonly found in tropical and subtropical regions, often referred to as the “Mycetoma belt.” This belt includes India, Sudan, Somalia, Yemen, Mexico, Senegal, Venezuela, Colombia, and Argentina. A few cases have been documented outside the traditional Mycetoma belt, such as Israel, Germany, the Netherlands, and Turkey [4].

The majority of cases occur in areas with hot and arid climates, where there is a short period of heavy rainfall and milder temperatures. Actinomycetoma, due to bacteria of the order Actinomycetales, is more widespread in arid regions, while eumycetoma, due to fungi, tends to occur in areas with higher rainfall [5].

Farmers, herders, and other persons engaged in outdoor work, particularly those who go barefoot, are frequently affected. Additionally, individuals with inadequate hygiene, malnutrition, diabetes, or severe immunodeficiency are also prone to such infections [6].

Bacteria of the genus *Gordonia* are types of actinomycetes that thrive in aerobic conditions. They test positive for catalase, and their Gram staining characteristics can vary from positive to variable. They are slightly acid-fast, nonmotile, and have a characteristic nocardioform (filamentous, branching) morphology. *Gordonia rubripertincta* colonies exhibit a striking bright orange or orange-red coloration [7].

Only a small number of human infections attributable to *Gordonia* species have been documented in the medical literature. Its identification poses significant diagnostic challenges due to its rarity, slow growth in culture, and similarity to other actinomycetes. *Gordonia* infections are most frequently identified in patients with medical devices, such as Hickman central venous catheters [8]. Infection of a sternal wound due to *Gordonia bronchialis* following a mitral valve replacement has also been described [9]. The first reported cases documenting catheter-related bloodstream infection caused by *Gordonia rubripertincta* occurred in dialysis patients [10].

*Gordonia* can cause infections in both immunocompetent and immunocompromised individuals [11]. *Gordonia* primarily causes respiratory tract infections in immunocompromised individuals. However, they can also cause other infections, such as catheter-related bloodstream, septic arthritis, endocarditis, central nervous system and soft tissue infections [7,8,9,10,11,12]. Five cases of *Gordonia* bacteremia were documented between 1999 and 2013. None of these cases involved *G. rubripertincta* [12].

There have been reported cases of actinomycetoma caused by other species of *Gordonia*, but *G. rubripertincta* has not previously been found (Table 2) [13,14,15]. A recent report by França et al. described a case of actinomycetoma due to *Gordonia soli*, highlighting the diagnostic challenges associated with this pathogen. The infection clinically resembled more common causes of mycetoma, with nodules, draining sinuses, and granule production, making microbiological confirmation essential. Molecular techniques such as 16S rRNA sequencing played a crucial role in establishing the correct diagnosis. The patient was treated with courses of linezolid plus meropenem and maintenance therapy with oral trimethoprim–sulfamethoxazole and ciprofloxacin [13]. Similarly, Gueneau et al. documented the first human mycetoma caused by *Gordonia westfalica*, reinforcing the expanding spectrum of *Gordonia* species capable of causing actinomycetoma. This case emphasized the importance of advanced laboratory identification methods, as conventional cultures may misidentify *Gordonia* as other actinomycetes or even as contaminants. 16S RNA sequencing after surgical biopsy identified *G. westfalica*. The patient received 4 weeks of oral trimethoprim–sulfamethoxazole and rifampicin. Complete response was observed, and antibiotic treatment was stopped. There was no relapse at 3 months [14]. Wang et al. reported an immunocompetent woman with actinomycetoma caused by *Gordonia terrae*, further demonstrating that host immunosuppression is not required for infection. Histopathology showed clumps of filamentous organisms and grains, the latter surrounded by matrix. Biopsy tissue culture grew Gram-positive *Gordonia terrae*, which was confirmed by 16S rRNA sequencing. The case illustrated the indolent and locally destructive nature of mycetoma, along with the need for prolonged antimicrobial therapy tailored to susceptibility results. The lesion was resolved after being treated with amoxicillin and clavulanic acid for 3 months [15].

Until 1989, *G. rubripertincta* was identified as *Rhodococcus rubropertinctus*. It was documented to have induced a lung infection clinically resembling tuberculosis in an immunocompetent individual who positively responded to oral anti-tuberculosis treatment. The patient did not exhibit any obvious signs of immunosuppression [16].

Clinically, mycetoma is identified by painless swelling, sinus tract formation, and discharge of granular material. Clinical variants include nodules without sinuses, cysts, and verrucous plaques [4]. The differential diagnosis of mycetoma includes Kaposi’s sarcoma, Charcot arthropathy, osteogenic sarcoma, and osseous tuberculosis [2,4,6]. Although the case was diagnosed as actinomycetoma caused by *Gordonia rubripertincta*, it should be noted that the clinical features may also be consistent with botryomycosis. Over 70 species of fungi and bacteria can cause mycetoma, with *Streptomyces somaliensis* and *Nocardia* species typically causing actinomycetoma, and *Madurella mycetomatis* most frequently causing eumycetoma [1,4,17].

The absence of sinus tracts in the observed case of actinomycetoma, despite the chronic nature of the disease over several years, may be attributed to several factors. Actinomycetoma, caused by *Nocardia* or *Actinomyces* bacteria, can present with firm nodules and a slowly progressing infection that does not always lead to the formation of sinus tracts. Additionally, the prolonged use of antibiotics could have influenced the clinical presentation. Prolonged antibiotic therapy may have slowed down the progression of the infection, potentially preventing the development of sinus tracts or reducing the extent of tissue damage. Furthermore, the immune response of the patient, as well as the location and severity of the infection, may contribute to the absence of external drainage pathways. Therefore, it is plausible that the combination of long-term infection and frequent antibiotic treatment could have played a role in the lack of sinus tracts in this case.

The negative microbiological results from bone biopsies further highlight the diagnostic difficulties previously reported in the literature, emphasizing the need for more sensitive molecular diagnostic tools and prolonged culture methods to improve detection of *Gordonia* species. The most accurate identification result can be obtained either by multilocus sequencing of housekeeping genes (16S rRNA, *gyr*B, and *sec*A) or by whole-genome sequencing [18], but these techniques are expensive and not always routinely available. We identified our isolate by the matrix-assisted laser desorption ionization–time of flight mass spectrometry (MALDI-TOF) method as *Gordonia rubripertincta* with high identification score as 2.25 (out of a maximum of 3.0).

Doppler ultrasound and sonography can be valuable in overcoming diagnostic challenges, such as negative bone biopsy results. Mycetoma, particularly eumycetoma, exhibits specific sonographic features and blood flow patterns that help differentiate between fungal and bacterial forms of the infection. Eumycetoma typically shows higher vascularity and more heterogeneous echotexture compared to actinomycetoma, making Doppler ultrasound a useful tool in diagnosing mycetoma when bone biopsies fail to provide conclusive results [19].

It is worth noting that while ELISA tests are available for certain actinomycetoma agents, such as *Nocardia brasiliensis*, their application remains limited to specific pathogens. Currently, diagnostic methods for actinomycetoma primarily rely on clinical presentation, histopathological examination, and microbiological cultures. Although ELISA tests offer a rapid and non-invasive approach for detecting *Nocardia brasiliensis* and potentially other actinomycetoma pathogens, their broader use is constrained by the availability of specific assays for other causative organisms. The development of more comprehensive diagnostic tools, including ELISA tests for a wider range of actinomycetoma-causing bacteria, would significantly improve diagnostic accuracy and facilitate more timely treatment in clinical practice [20].

Treatment for actinomycetoma involves antibiotics like amikacin, dapsone, trimethoprim–sulphamethoxazole, and streptomycin sulfate. Resistant cases may require carbapenems, amoxicillin–clavulanic acid, clindamycin, or quinolones. Persistent cases may require surgical intervention, from local excision and drainage to amputation [2,4,21,22].

Mycetoma infections pose several treatment challenges. They often persist for years, requiring prolonged therapy. The case illustrates that standard antibiotic treatments may not always yield positive outcomes. The initial empirical treatment was ineffective, emphasizing the necessity of targeted antibiotic therapy guided by antimicrobial susceptibility testing and consideration of tissue penetration.

The effective management of this challenging condition necessitates a multidisciplinary approach involving orthopedic surgeons, ID specialists, radiologists, microbiologists, dermatologists, pathologists and at times, plastic surgeons. This case illustrated the diagnostic and treatment challenge of mycetoma where the disease is not endemic.

This clinical case has a limitation: the culture was not preserved for imaging the test results, although it is clearly depicted in the literature, and the clinical isolate was not deposited in a recognized culture collection.

## 4. Conclusions

The findings of this case are consistent with the existing literature, which describes *Gordonia* infections as rare and typically opportunistic, often affecting immunocompromised patients. However, this case is notable for involving an immunocompetent patient who developed a chronic, progressive infection, which is less commonly reported in the literature. While cases of actinomycetoma caused by *Gordonia* species have been documented, to the best of our knowledge, this is the first reported case of actinomycetoma specifically caused by *Gordonia rubripertincta*.

## Figures and Tables

**Table 1:** MIC of *Gordonia rubripertincta*.

Antimicrobial Agent	MIC
Penicillin	0.125 mg/L
Amoxicillin–clavulanate	0.016 mg/L
Ciprofloxacin	0.047 mg/L

**Table 2:** Reported cases of actinomycetoma caused by *Gordonia* species.

Author, Year	Country	*Gordonia* Species	Clinical Presentation	Diagnostic Methods	Treatment	Clinical Outcome
França et al., 2023 [13]	Brazil	*Gordonia soli*	Nodules, draining sinuses, and granule production	Culture; 16S rRNA gene sequencing	Linezolid + meropenem, followed by oral trimethoprim–sulfamethoxazole and ciprofloxacin	Clinical improvement reported
Gueneau et al., 2020 [14]	France	*Gordonia westfalica*	A suppurative and nodular swelling of the first interdigital space, with several discharging sinuses	Surgical biopsy; culture; 16S rRNA gene sequencing	Oral trimethoprim–sulfamethoxazole + rifampicin for 4 weeks	Complete response; no relapse at 3 months
Wang et al., 2018 [15]	China	*Gordonia terrae*	A slowly growing mass on her left foot	Histopathology; culture; 16S rRNA gene sequencing	Amoxicillin–clavulanic acid for 3 months	Complete resolution
Siksniute et al., 2026, present case	Lithuania	*Gordonia rubripertincta*	Deformation, pain, and severe swelling over the medial, dorsal and plantar aspects of the foot	Culture; MALDI-TOF MS	After various antibiotic regimens, ampicillin–sulbactam + ciprofloxacin for 6 weeks	Clinical improvement

## Data Availability

The raw data supporting the conclusions of this article will be made available by the authors on request.
